# Impact of Noise on Molecular Network Inference

**DOI:** 10.1371/journal.pone.0080735

**Published:** 2013-12-05

**Authors:** Radhakrishnan Nagarajan, Marco Scutari

**Affiliations:** 1 Division of Biomedical Informatics, Department of Biostatistics, University of Kentucky, United States of America; 2 UCL Genetics Institute, University College London, London, United Kiingdom; Leibniz-Institute for Farm Animal Biology (FBN), Germany

## Abstract

Molecular entities work in concert as a system and mediate phenotypic outcomes and disease states. There has been recent interest in modelling the associations between molecular entities from their observed expression profiles as networks using a battery of algorithms. These networks have proven to be useful abstractions of the underlying pathways and signalling mechanisms. Noise is ubiquitous in molecular data and can have a pronounced effect on the inferred network. Noise can be an outcome of several factors including: inherent stochastic mechanisms at the molecular level, variation in the abundance of molecules, heterogeneity, sensitivity of the biological assay or measurement artefacts prevalent especially in high-throughput settings. The present study investigates the impact of discrepancies in noise variance on pair-wise dependencies, conditional dependencies and constraint-based Bayesian network structure learning algorithms that incorporate conditional independence tests as a part of the learning process. Popular network motifs and fundamental connections, namely: (*a*) common-effect, (*b*) three-chain, and (*c*) coherent type-I feed-forward loop (FFL) are investigated. The choice of these elementary networks can be attributed to their prevalence across more complex networks. Analytical expressions elucidating the impact of discrepancies in noise variance on pairwise dependencies and conditional dependencies for special cases of these motifs are presented. Subsequently, the impact of noise on two popular constraint-based Bayesian network structure learning algorithms such as Grow-Shrink (GS) and Incremental Association Markov Blanket (IAMB) that implicitly incorporate tests for conditional independence is investigated. Finally, the impact of noise on networks inferred from publicly available single cell molecular expression profiles is investigated. While discrepancies in noise variance are overlooked in routine molecular network inference, the results presented clearly elucidate their non-trivial impact on the conclusions that in turn can challenge the biological significance of the findings. The analytical treatment and arguments presented are generic and not restricted to molecular data sets.

## Introduction

Identifying associations and network structures from observational data sets obtained across a given set of entities is a challenging problem and of great interest across a spectrum of disciplines including molecular biology [1]–[8]. While the molecular entities of interest are represented by the *nodes*, their associations are represented by the *edges*. Such networks can prove to be convenient abstractions of the underlying pathways and signalling mechanisms across distinct phenotypes and disease states. [1], [2], [7]. They can reveal interesting characteristics including repetitive structures, dominant players, community structures and generative mechanism [9]–[11] that can assist in developing meaningful interventions.

Molecular data obtained from biological systems may or may not have explicit temporal information. While the former explicitly captures the evolution of the molecular activity as a function of time (*dynamic*), the latter represents a snapshot of the biological activity in a given window of time (*static*). Dynamic data sets are rare and challenging to generate since they demand controlling a number of factors. Static data sets in conjunction with multiple independent realizations are relatively easier to generate. Their prevalence may also be attributed to the tradition of generating replicate measurements in molecular biology in order to demonstrate reproducibility of the findings. Prior studies on static data sets used pairwise dependency measures to capture the associations between a given set of molecules in the form of *relevance networks* [1]. The underlying hypothesis being that correlated genes are likely to be co-regulated or functionally related [12]. However, pairwise dependency measures by definition are symmetric measures resulting in *undirected graphs*. It is also known that the dependency between a given pair of genes may not necessarily be direct and possibly mediated by other gene(s). This possibly motivated the choice of conditional dependencies as opposed to pairwise dependencies for molecular network inference. Subsequently, probabilistic approaches such as Bayesian network structure learning techniques that model the conditional dependencies across a larger number of variables in an automated manner were proposed to infer molecular networks from static data sets [3], [6], [7]. The resulting networks of constraint-based structure learning are typically in the form of *directed acyclic graphs* (DAGs) or *partially directed acyclic graphs* (PDAGs). While DAGs have directed edges, PDAGs have directed as well as undirected edges and accommodate the presence of *equivalent classes* [13], [14]. Constraint-based structure-learning algorithms by their very nature do not accommodate the presence of cycles and feedback between the molecules of interest which is an inherent limitation. They have nevertheless proven to be useful approximations of pathways and signalling mechanisms [6], [7], [13]. The DAGs (PDAGs) may also reveal possible *causal relationships* between the nodes under certain implicit assumptions [15].

Of interest, is to note that these molecular data sets are inherently noisy [16], [17], [18]. Noise and its variation across molecular entities may have contributions from several factors including stochastic mechanisms coupled to the systems dynamics, sensitivity and precision of the measurement device, variations in abundance of specific molecules, preferential binding affinities and experimental artefacts that are an outcome of the estimation process [7], [19], [20], [21]. While identifying the source of noise is a challenging problem in its own merit, understanding its impact on network inference procedure is especially critical in order to avoid identification of spurious associations. In a recent study, we elucidated the non-trivial impact of noise and auto-regulatory feedback on networks inferred using Granger causality tests. The results were established on multivariate time series generated using gene network motifs modelled as vector auto-regressive processes (VAR) [22], as well as those inferred from cell-cycle microarray temporal gene expression profiles [23], [24]. The present study investigates the impact of noise on pair-wise correlation, partial correlation and constraint-based structure learning algorithms by considering static data sets generated from linear models of popular *network motifs* and publicly available molecular expression data [7]. Network motifs are repetitive atomic structures that have been found to be prevalent across more complex networks [9]. In the present study, we consider three popular three-node motifs, namely: *common-effect, three-chain* and the *coherent type-I feed-forward loop (FFL)* [9], [25], [26]. The *common-effect motif* and the *three-chain motif* represent the *convergent* and *serial connection* respectively. These connections comprise the *fundamental connections* in Bayesian networks [27]. Furthermore, the conditional independence relationships represented by these motifs are usually among the first to be examined in any constraint-based structure learning algorithm justifying their choice. Common-effect motif is also an essential ingredient in identifying *equivalent classes* and PDAGs [13]. The coherent type-I FFL has been shown to persist across a number of organisms including E. Coli and S. Cerivisiae [25], [26]. Of interest, is to note that three-chain and common-effect motifs are an integral part of a type-I coherent FFL. Analytical expressions for large discrepancies in noise variance on pairwise (*correlation coefficient*) and conditional dependencies (*partial correlation*) are investigated. The impact of such discrepancies on constraint-based Bayesian network structure learning is also investigated. Finally, the presence of significant discrepancies in noise variance and its impact on network inference from experimental molecular expression profiles [7] is investigated.

## Methods and Results

Prior to investigating the impact of noise on the constraint-based Bayesian network structure learning algorithms, its impact on pairwise and conditional dependencies across the three network motifs is investigated.

### 2.1 Pairwise and Conditional Dependencies

#### Network Motif Parameters

In the following discussion, 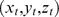 represent the molecular expression of the three genes 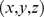 respectively in a small time window 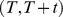. The terms 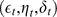 represent zero-mean, unit-variance uncorrelated noise attributed to inherent uncertainties and artifacts prevalent in molecular expression studies. Parameter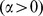 represents the transcriptional coupling strengths between the genes and is constrained to be equal across the genes, since the impact of variations in 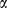 on pairwise and conditional dependencies is expected and not the goal of the present study. Discrepancies in the noise variances across the nodes are represented by parameters 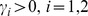.

#### Case 1: Common-effect network motif

**Figure 1:**
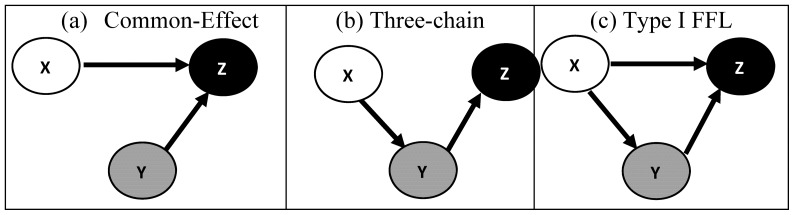
Popular three-gene network motifs: common-effect, three-chain and coherent type-I feed-forward loop are shown in (a), (b) and (c) respectively.

The common-effect network motif (*v*-structure) [13] is a fundamental connection, Fig. 1a, discussed widely within the context of Bayesian network structure learning algorithms. For this motif, 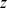 is regulated by 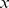 and 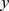 given by the linear model,
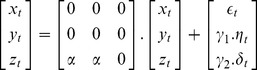
(1)


The correlation coefficients are given by
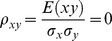





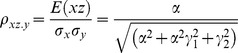


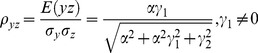
(2)


The partial correlations are given by
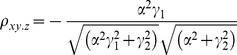


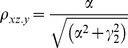


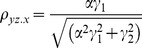
(3)


For large noise limit at z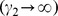 with finite noise at y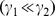, the correlation coefficients are given by
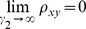


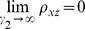


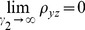
(4)


The partial correlations are given by
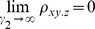


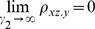


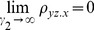
(5)


For large noise limit at y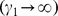 with finite noise at z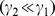, the correlation coefficients are given by
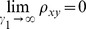


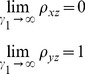
(6)


The partial correlations are given by
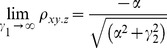


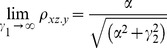


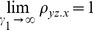
(7)


#### Remark 1

*Correlation coefficient estimates reveal significant pairwise dependencies across (* 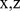 *) and (* 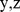 *) in contrast to (* 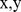 *) resulting in the undirected graph* 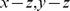 *. As expected, conditioning the marginally independent nodes* 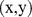 *on* 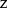 *renders them dependent* 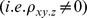.

- *(i)Large noise limit at the common-effect node* 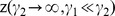 *: Pairwise as well as conditional dependencies vanish (4, 5) challenging any reliable conclusion on the network structure in the large noise limit when* 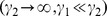 *preventing any reliable inference of the network. More importantly, conditioning on the common-effect node at large noise levels did not render* 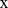 *and* 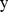 *dependent as expected (5).*
- *(ii)Large noise limit at one of the causes* 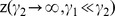 *: Pairwise dependencies (* 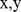 *) as well as (* 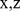 *) disappear (6). Interestingly, conditional dependencies* 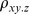 *and* 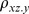 *are equal in magnitude with opposite signs and function of* 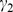 *(7). Pairwise as well as conditional dependencies* 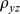 *and* 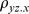 *have maximal values of unity in the large noise limit at* ***y*** *.*

#### Case 2

*Three-chain network motif*

Consider the three-chain network motif [9], Fig. 1b, where 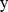 mediates the activity between 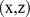 given by the linear model
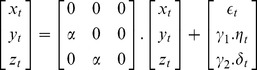
(8)


The correlation coefficients are given by
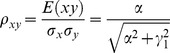


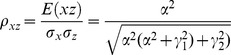


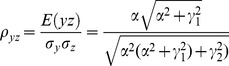
(9)


The partial correlations are given by
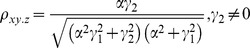


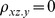


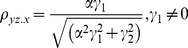
(10)


For large noise limit at 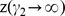 with finite noise at 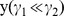, the correlation coefficients are given by
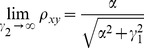


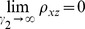


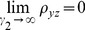
(11)


The partial correlations are given by
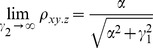


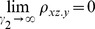


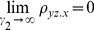
(12)


For large noise limit at 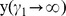 with finite noise at 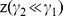, the correlation coefficients are given by
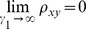


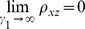


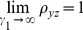
(13)


The partial correlations are given by
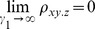


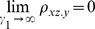


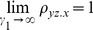
(14)


#### Remark 2

*Correlation coefficient estimates reveal significant pairwise dependencies across (* 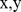 *), (* 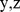 *) and (* 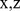 *) resulting in the undirected graph* 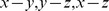 *. As expected, conditioning the marginally dependent nodes* 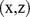 *on* 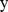 *renders them independent* 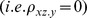 *. This result is immune to the choice of the linear model parameters and reflects possible directed acyclic graph of the form* 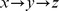 *.*

- *(i). Large noise limit at the node* 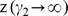 *: Pairwise dependencies (11),* 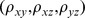 *are identical to the conditional dependencies in (12),* 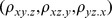 *. Of interest is to note that pairwise dependencies* 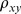 *and conditional dependency* 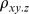 *have identical non-zero magnitude.*
- *(ii). Large noise limit at the node* 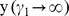 *: Pairwise dependencies (13),* 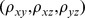 *are identical to those of conditional dependencies (14),* 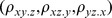 *similar to what was observed for* 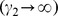 *. However, in contrast to* 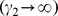 *, pairwise* 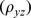 *and conditional dependencies* 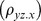 *are identical with a maximum value similar to that of the common-effect network motif. Also, pair-wise dependencies* 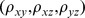 *(13) are identical to those obtained for the common-effect motif (6) failing to distinguish these two structures.*

#### Case 3

*Coherent Type-I feed-forward loop network motif*

Consider the coherent type-I feed-forward loop [25], [26], Fig. 1c, where the expression of 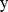 is regulated by 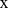 whereas those of 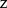 is regulated by 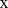 as well as 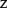 given by the linear model
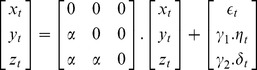
(15)


The correlation coefficients are given by
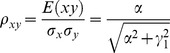


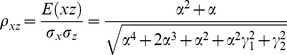


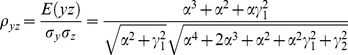
(16)


The partial correlations are given by
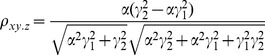


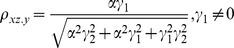


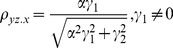
(17)


For large noise limit at 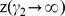 with finite noise at 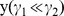, the correlation coefficients and partial correlations are given by
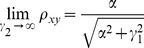


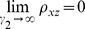


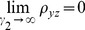
(18)


The partial correlations are given by
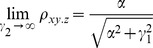


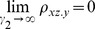


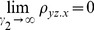
(19)


For large noise limit at 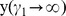 with finite noise at 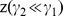, the correlation coefficients and partial correlations are given by
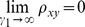


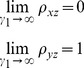
(20)


The partial correlations are given by
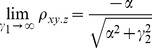


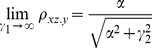


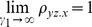
(21)


#### Remark 3

*Correlation coefficient estimates reveal significant pairwise dependencies across (* 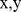 *), (* 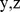 *) and (* 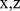 *) indicating a possible undirected graph of the form* 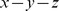 *. Unlike the three-chain, conditioning* 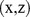 *on* 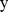 *does not render them independent.*

- *(i). Large noise limit at* 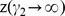 *: Pairwise dependencies (18) and conditional dependencies (19) are identical to those obtained for the three-chain motif (11, 12) failing to distinguish these structures for relatively large noise variance at* 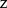, *Table 1* *.*
- *(ii). Large noise limit at* 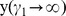 *: Pairwise dependencies (20) and conditional dependencies (21) are identical to those obtained for the common-effect motif (6), (7) failing to distinguish these structures for relatively large noise variance at* 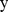 *,* *Table 1* *. Also, the pairwise dependencies for* 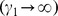 *is identical for the coherent Type I FFL, three-chain as well as the common-effect motif.*

**Table 1:** Pair-wise and conditional dependencies across the three network motifs in the asymptotic noise limits.

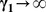	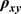	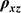	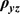	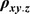	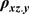	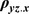
Common-Effect	0	0	1	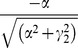	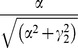	1
Three-Chain	0	0	1	0	0	1
Type I FFL	0	0	1	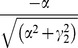	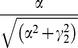	1
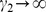	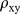	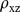	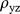	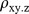	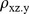	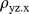
*Common-Effect*	0	0	0	0	0	0
*Three-Chain*	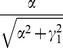	0	0	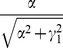	0	0
*Type I FFL*	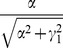	0	0	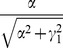	0	0

### 2.2 Constraint-based Bayesian Network Structure Learning

Bayesian network structure learning algorithms have been used successfully to infer the associations between a large numbers of variables. Several such algorithms have been proposed in literature, a partial list of contributions include [28], [29], [30], [31], [32]. In the present discussion, we focus on constraint-based structure learning algorithms that infer the network structure using tests for conditional independence, namely: the Grow-Shrink (GS) algorithm [30] and the Incremental Association Markov Blanket (IAMB) [31].

GS was the first algorithm that learned the *Markov blanket* of each node as an intermediate step to speed up structure learning process. The Markov blanket 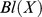 of a node 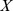 is defined as the set of nodes that makes 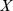 independent from all the other nodes in the domain. In a Bayesian network, it is formed by the parents of 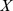, its children, and the other parents of its children [15]. Therefore, the search for the neighbors of each node can be restricted to its Markov blanket, which in most cases contains a limited number of nodes. GS learns Markov blankets using a forward selection (*Growing Phase*) followed by a backward selection (*Shrinking Phase*). Conditional independence tests are performed in order of increasing complexity (i.e. with respect to the number of nodes involved in the test) in order to maximize the overall power of the structure learning algorithm. Markov blankets are then reduced to the corresponding set of neighbors by an additional backward selection. Arc directions are established starting from *v*-structures, which can be identified by the interplay of the causes conditional on their common effect, and then propagated to prevent the formation of further *v-*structures and enforce *acyclicity*. This is achieved using the heuristics described elsewhere [30], [33]. IAMB introduces relatively better heuristics to identify Markov blankets while improving on GS by using a forward stepwise regression. However, IAMB in contrast to GS is designed to identify the Markov blanket of each node and not the complete network structure. Essentially, it performs the same task as the first step of GS but the forward stepwise selection in IAMB reduces the number of nodes incorrectly included in the Markov blankets. In the context of Bayesian network structure learning, IAMB is extended to a complete learning algorithm by adding steps 2 to 4 of GS. While both algorithms have been shown to be formally correct, IAMB has been recently supported by more extensive proofs and simulations [34], [35]. Of interest is to note that GS as well as IAMB are highly dependent on the ability of the conditional independence tests to correctly identify dependence relationships. In fact, the proofs of correctness of both structure learning algorithms implicitly assume absence of type I or type II errors. Such an assumption can especially be violated in the presence of noise that may accentuate false-positives as well as false-negatives challenging the biological significance of the results. This in turn justifies investigating the impact of discrepancies in noise variance across the nodes on network inference using GS and IAMB. Since the conditional independence tests increase in complexity during the structure learning process across GS and IAMB [36] the present study is restricted to well-established network motifs that are prevalent across more complex structures. The concerns presented across these motifs are expected to be aggravated across more complex network topologies.

### Common-effect network motif

*For large noise limit at* 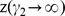 *with finite noise at* 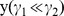 *:*

For relatively large noise variance at 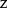, the pairwise as well as conditional dependencies (4, 5) vanish across GS as well as IAMB resulting in an empty network. This happens regardless of the values of 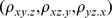 because both GS and IAMB test for significant pairwise dependencies 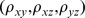 first and conclude the Markov blankets of 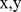 and 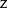 to be empty sets. As a consequence, none of the nodes have any neighbours resulting in an empty graph.

*For large noise limit at* 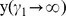 *with finite noise at* 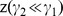 *:*

For relatively large noise variance at 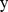, GS was able to retrieve a part of the network structure as discussed below. The Markov blankets inferred by GS are as follows:

- *For* 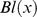 *from (6) we have* 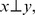 *i.e.* 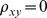 *and* 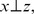 *i.e.* 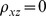 *resulting in* 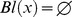 *.*
- *For* 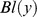 *, from (6) we have* 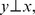 *i.e.* 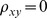 *and* 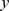 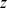 *, i.e.* 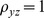 *. As a result,* 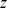 *is added to* 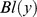 *. Also from (7),* 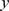 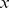 *given* 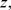 *i.e.* 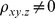 *since* 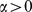 *. Therefore,* 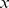 *is added to* 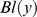 *for suitable values of* 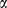 *resulting in* 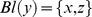 characteristic of the motif (1)*.*
- *For* 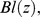 *from (6) we have* 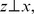 *i.e.* 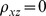 *but* 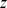 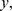 *i.e.* 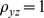 *. As a result,* 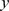 *is added to* 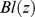 *. Also from (7)* 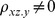 *, since* 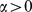 *. Therefore, a suitable choice of* 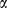 *results in the Markov blanket* 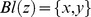 *characteristic of the motif (1).*

For IAMB, the conditional independence tests are performed in a different order since the nodes are included in the Markov blankets in decreasing order of association. However, the resulting Markov blankets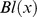, 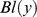 and 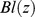 are same as those of GS. The impact of discrepancies in noise variance across the nodes on structure learning is especially elucidated by the asymmetry of the Markov blankets 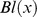 and 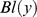 as well as 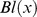
and 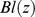. Markov blankets are symmetric by definition, i.e.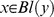 then 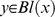 and vice versa. However, for the present case we have following asymmetries (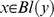 while 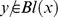) and (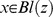 while 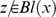) violating the definition of Markov blanket. For consistency, a *symmetry correction* [34], [35] may be applied either by removing 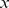 from 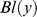 and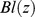, or adding 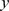 and 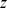 to 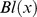. The latter correction enables faithful reproduction of the motif while the former does not.

### Three-Chain network motif

*For large noise limit at* 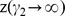 *with finite noise at* 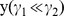 *:* 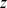


For relatively larger noise variance at 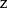, the Markov blankets inferred by GS are given as follows:

- *For* 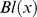 *, from (11) we know that* 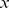 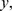 *i.e.* 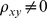 *since* 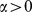 *. For suitable choice of* 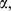 *we may correctly infer* 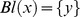 *. Also, from (11, 12) we have* 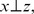 *i.e.* 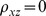 *and* 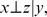 *i.e.* 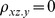 *so* 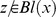 *. Therefore, the ability to infer* 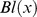 *depends on* 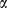 *.*
- *For* 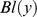 *, from (11, 12) we have* 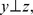 *i.e.* 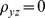 *and* 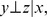 *i.e.* 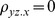 *resulting in either* 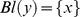 *or* 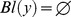 *markedly different from* 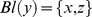 characteristic of the motif (8)*.*
- *For* 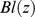 *, from (11) we have* 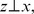 *i.e.* 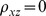 *and* 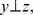 *i.e.* 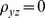 *resulting in* 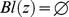 *in contrast to* 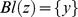 *characteristic of the motif (8).*

As in the case of common-effect network motif, reordering of the conditional independence tests in IAMB does not result in Markov blankets different from those inferred by GS. Unlike common-effect motif, no asymmetry between the Markov blankets is observed for the three-chain, since 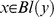 and 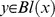 are established using the same correlation coefficient 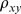. Given these set of Markov blankets, identifying the correct network structure is impossible. Since for large values of 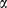, both GS and IAMB learn (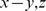), while for small values of 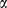 both GS and IAMB are unable to identify any of the arcs present in the true motif structure. The presence of at most a single arc 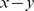 makes it impossible to infer its direction, since both GS and IAMB use *v*-structures to infer directions and the learned motif structure contains none.

*For large noise limit at* 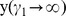 *with finite noise at* 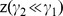 *:*

For relatively large noise variance at 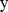, no reliable conclusion of the motif is possible across GS as well as IAMB. The Markov blankets are as follows:

- *For* 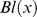 *, from (13) we have* 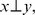 *i.e.* 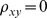 *and* 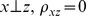 *. As a result,* 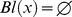 *in contrast to* 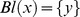 *characteristic of the motif (8).*
- *For* 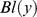 *, from (13) we have* 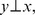 *i.e.* 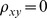 *but* 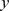 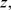 *i.e.* 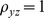 *. Even after updating the Markov blanket to* 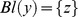 *, the dependence between x and y is obscured by noise as* 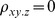 *. Therefore, the Markov blanket* 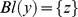 *.*
- *For* 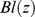 *, from (13) we have that* 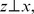 *i.e.* 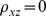 *but* 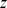 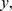 *i.e.* 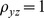 *. Also, from (14) we have* 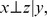 *i.e.* 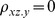 *. This results in the Markov blanket* 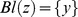 *characteristic of the motif (8).*

In this case, no asymmetry is observed despite the effects of noise. Nevertheless, neither GS nor IAMB was able to learn the motif for relatively large noise variance.

### Coherent Type-I Feed-Forward Loop motif

*For large noise limit at* 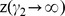 *with finite noise at* 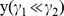 *:*

For relatively large noise variance at 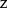, the Markov blankets determined by GS and IAMB are as follows:

- *For* 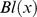 *, from (18),* 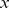 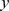 *i.e.* 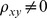 *, since* 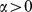 *. Also from (18, 19) we note that* 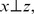 *i.e.* 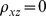 *and* 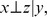 *i.e.* 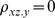 *. Therefore,* 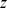 *is not included in* 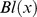 *. Thus, GS and IAMB return either* 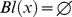 *or* 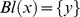 *for suitable choice of* 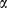 *in contrast to* 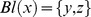 *characteristic of the motif (15).*
- *For* 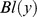 *, from (18)* 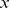 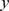 *, i.e.* 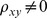 *, since* 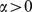 *. Also, from (18, 19) we have* 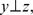 *i.e.* 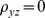 *and* 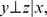 *i.e.* 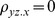 *. Therefore,* 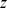 *is not included in* 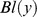 *. Thus, GS and IAMB return either* 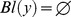 *or* 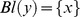 *for suitable choice of* 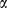 *as opposed to* 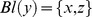 characteristic of the motif (15).
- *For* 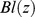 *, it is impossible to learn the correct Markov blanket* 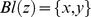 *since* 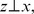 *i.e.* 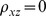 *as well as* 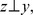 *i.e.* 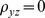 f*rom (18). As a result,* 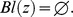

In the present case, discrepancy in noise variance does not result in asymmetry in the Markov blankets. Thus, symmetry correction may not alleviate the impact of noise. Possible motif structures corresponding to large discrepancies at 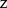 are either an empty structure or 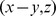. This is problematic for two reasons. First, only one arc out of three is correctly identified and its direction cannot be determined by the learning algorithm. Second, the motif structures above are indistinguishable from those obtained for the three-chain network motif.

*For large noise limit at* 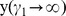 *with finite noise at* 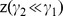 *:*

For relatively large noise variance 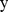, again neither GS nor IAMB was able to infer the motif. The Markov blankets are given as follows:

- *For* 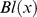 *, from (20) we have* 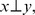 *i.e.* 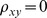 *and* 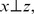 *i.e.* 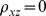 *. This results in Markov blanket* 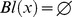 *in contrast to* 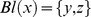 *For* 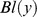 *, from (20) we have* 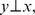 *i.e.* 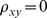 *. However,* 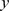 *is dependent on* 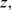 *i.e.* 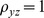 *. Also, from (21) we have* 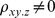 *, since* 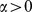 *. This results in Markov blanket* 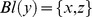 *characteristic of the motif (15) for suitable choice of parameter* 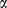 characteristic of the motif (15).
- *For* 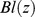 *, from (20) we have* 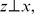 *i.e.* 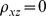 *. However,* 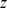 *is dependent on* 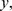 *i.e.* 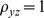 *. Also, from (21)* 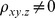 *, since* 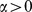 *. These results in turn result in* 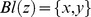 *for suitably large values of* 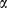 *.*

Asymmetry between the Markov blankets is observed across 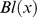 and 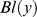 as well as between 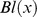 and 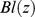. This can be attributed to the fact that 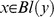 while 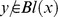 and 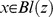 while 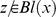 for suitably large values of 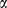. Correcting this asymmetry by adding 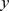 and 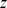 to 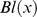 results in the Markov blankets characteristic of the motif. However, establishing their directions is not possible since the presence of an arc between 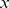 and 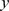 prevents both GS and IAMB from identifying 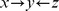. As a result, all possible configurations of the arcs' directions are probabilistically equivalent resulting in an undirected graph. This is phenomenon is known as the *shielded collider* identification problem and affects all constraint-based learning algorithms [37].

### 2.3 Simulation Results

In the following discussion, the three gene network motifs are generated using (1, 8, 15) with parameter 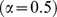 and normally distributed noise. Since the objective is to demonstrate the impact of noise as opposed to the other parameter,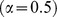, is held constant across all the simulations. The noise variance at the node 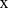 is fixed at unit variance whereas those at 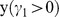 and 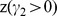 are varied systematically in order to understand the impact of discrepancy in noise variance on the conclusions. Three distinct cases of noise variances, namely: 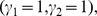 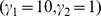 and 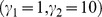 are considered. The cases 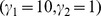 and 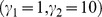 correspond to large noise variance limits as discussed under (Cases 1, 2 and 3) whereas 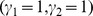 corresponds to absence of discrepancies in noise variance. The conditional independence tests used in the following discussion is exact t-test for Pearson correlation as implemented in the R package bnlearn [38]. A description of the functions in bnlearn can be found in the accompanying manual with applications to molecular expression profiles in [39].

Results generated using constraint-based structure learning algorithms GS and IAMB were quite similar consistent with their expected behaviour, Section 2.2. Therefore, we discuss only the results from the GS algorithm. The networks were learned across 200 independent realizations of the data (sample size  = 2000) and Friedman's *confidence* 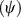 [3] was computed for each of the edges. Friedman's confidence essentially represents the percentage of times an edge shows up across networks learnt independently from bootstrapped realizations. In the case of observational data sets, confidences are estimated from networks learned from nonparametric bootstraps of the given empirical sample. In the present study, the underlying model generating the networks is known a priori. Therefore, parametric bootstrap is used where independent realizations of the data were generated from the model in contrast to non-parametric bootstrap [40]. Also, in the present study, confidence estimates of edges known to be present in the given graph a priori essentially represent their *statistical power*. As a rule of thumb [3], edges with confidence at least 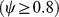 were deemed significant. In a recent study [41], we proposed a noise floor approach in order to avoid the ad-hoc choice of 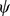, and subsequently a statistically motivated approach that estimates optimal 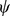 from the cumulative distribution of the confidence values [42]. However, in the present study the actual confidence values are presented for enhanced clarity.

#### Common-effect network motif

**Figure 2:**
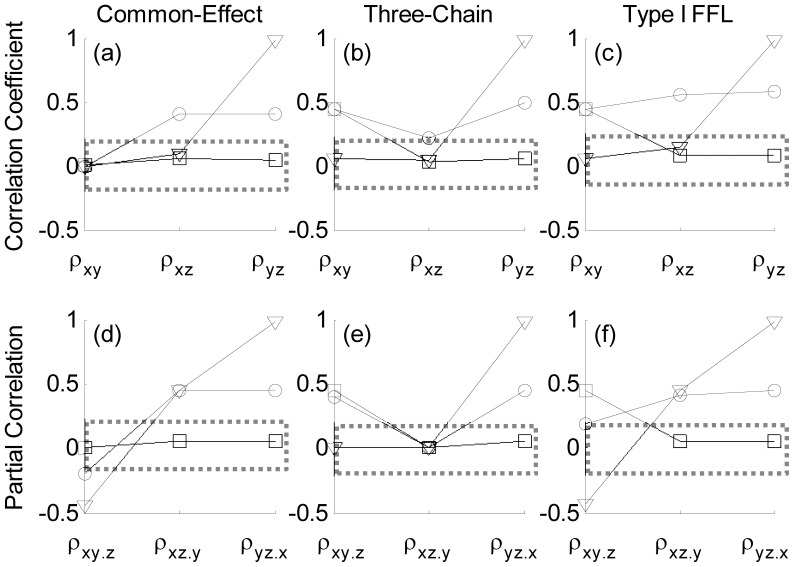
The average correlation coefficient and partial correlation estimates across 200 independent realizations of the common-effect, three-chain and coherent Type I feed-forward loop network motifs for various choices of noise variances 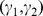 are shown in (a, d), (b, e) and (c, f) respectively. The *x*-axis labels correspond to the correlation coefficients 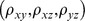 in (a, b, c) and partial correlations 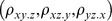 in (d, e, f) respectively. The (circles, squares and triangles) in each of the subplots correspond to noise variances with magnitudes 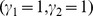, 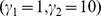 and 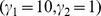 respectively. The points bounded by the dotted rectangle represent cases that occurred much lesser than 80% of the time as significant (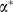 = 0.001) across 200 independent realizations.

**Figure 3:**
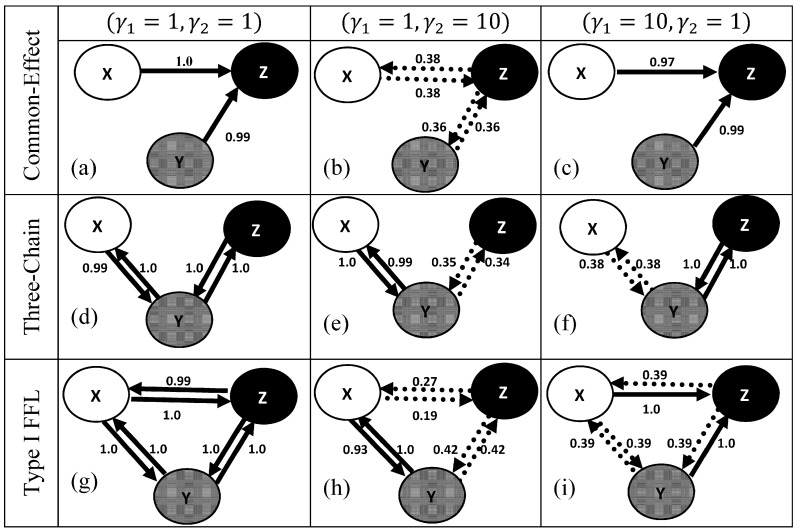
Bayesian networks inferred using Grow-Shrink algorithm along with Pearson correlation (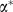 = 0.01) for the three-gene network motifs, namely: common-effect (a-c), three-chain (d-f) and coherent type-I feed-forward loop (g-i) for various choices of noise variances: 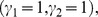 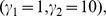 and 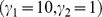. The confidences of the edges 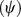 are represented as percentage of the edges that persisted across 200 independent realizations. Edges with 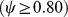 are shown by solid arrows whereas others 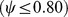 are shown by dotted arrows. Edges with confidence 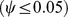 are deemed noisy and excluded for clarity.

The common-effect network motif, Fig. 1a, was generated using (1) with 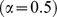 and normally distributed noise 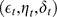. For finite and equal noise variance 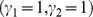 at 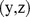 the correlation coefficients 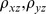 were similar and relatively higher than 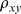 (∼0) as expected (2), Fig. 2a. In order to investigate the impact of large discrepancies in the noise variances, the noise variance across 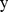 was increased relative to 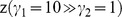. This resulted in small values of 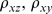 relative to 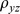, Fig. 2a and resembled (6) as expected. A similar analysis with 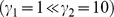 across 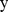 and 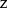 resulted in small correlation coefficients across the board similar to (4), Fig. 2a. Therefore, large discrepancies in noise variances across the nodes can have a pronounced effect on the pair-wise dependencies. The corresponding partial correlations for the three choices of noise variance 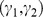 are shown in Fig. 2d. For finite equal noise variance 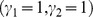 at 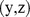, the partial correlation 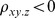 (3) was non-zero in contrast to 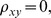 rendering the marginally independent nodes 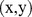 dependent. Increasing the noise variance across 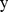 relative to 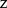 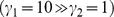 resulted in a significant increase in 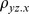 (7) whereas for 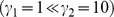, all the conditional dependencies were rendered negligible (5) preventing any reliable conclusion of the network structure, Fig. 2d. For finite equal noise variance 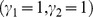 at 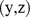, GS was able to faithfully retrieve the structure of the common-effect motif, Fig. 3a. Increasing the noise variance across 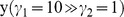 relative to 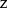, also retrieved the structure faithfully, Fig. 3c. However, increasing the noise variance on the common effect node 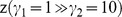 resulted in low confidence values of the edges challenging any reliable inference of the network, Fig. 3b. Thus the magnitude of the noise variance at the nodes can have a pronounced effect on constraint-based structure learning of a common-effect network motif.

#### Three-chain network motif

The three-chain network motif, Fig. 1b, was generated using (8) with 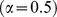 and normally distributed noise 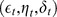. For finite and equal noise variance 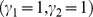 at the nodes 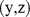
the correlation coefficients 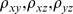 were significant as expected (9) with 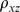 representing the transitive dependency between 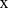 and 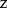, Fig. 2b. In order to investigate the impact of large noise variance, the noise variance on the mediating node 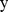 was increased relative to 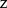 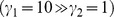. This resulted in small values of 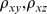 relative to 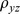 (13) similar to what was observed for the common-effect network motif (6) failing to distinguish these structures. On the other hand, large noise variance on the terminal node 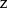 relative to 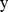 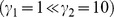 resulted in 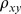 values relatively higher than that of 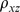 and 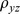, as expected from Fig. 2b. These results clearly demonstrate the non-trivial impact of noise strengths on network inference on pairwise dependencies. Partial correlations 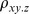 and 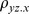 for finite equal noise variance 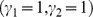 were considerably higher than that of 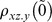 as expected, since conditioning on the mediator 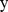 should render marginally dependent nodes 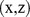 independent. Increasing the noise variance at 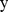 relative to 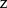 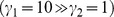 and at 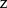 relative to 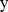 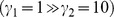, rendered the pairwise and conditional dependencies similar. This is reflected by the similar profiles, Figs. 2b and 2e respectively. For finite equal noise variance 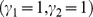 at 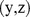, GS was able to faithfully retrieve the underlying undirected graph, Fig. 3d. This is to be expected since the Markov equivalent structure of the three-chain network motif is the undirected graph 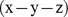. Increasing the noise variance across the mediator 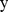 relative to 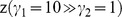 resulted in low confidence along 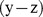 preventing any reliable inference of possible association between these nodes, Fig. 3e. Interestingly, increasing the noise variance on the terminal node 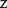 relative to 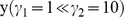 resulted in low confidence along 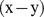 preventing any reliable inference of possible association between these nodes, Fig. 3f.

#### Coherent Type-I feed-forward loop network motif

The coherent Type-I feed-forward loop network motif, Fig. 1c, was generated using (15) with 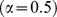 and normally distributed noise 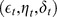. While one part of the Type-I FFL resembles the common-effect motif 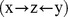, the other part resembles a three-chain 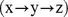, Fig. 1b. For finite and equal noise variance 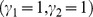 at 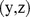 the pairwise (16) and conditional dependencies (17) were non-zero. Increasing the noise variance across 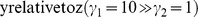 resulted in pairwise (20) identical to those of the common-effect (6) and three-chain motifs (13) failing to distinguish these network structures. This is reflected by similar profiles across Figs. 2a, 2b and 2c. On a related note, increasing the noise variance across 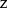 relative to 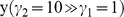 resulted in pairwise (18) and conditional dependencies (19) identical to those of the three-chain motif (11, 12) failing to distinguish these two distinct network structures. Similarities in the pairwise and conditional dependencies across these motifs are also reflected by similar profiles between Figs. 2b and 2c and between Figs. 2e and 2f respectively. For finite equal noise variance 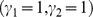 at 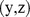 GS was able to retrieve the undirected edges 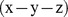, Fig. 3g. Failure to retrieve the exact structure, Fig. 1c, can be attributed to the presence of equivalent classes. Increasing the noise variance across 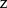 relative to 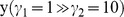 resulted in low confidences along 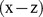 and 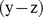 relative to 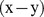 preventing any reliable inference of possible associations along 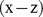 and 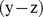, Fig. 3h. Thus for these choices of noise variances it is possible the results of GS for Type 1 FFL resembles the structure of the three-chain failing to distinguish them. In contrast, increasing the noise variance at 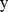 relative to 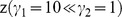 resulted in large edge confidence only along 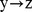 and 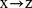 with low edge confidence along 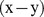 Fig. 3i preventing any reliable inference of the network structure.

### 2.4 Application to Molecular Expression Profiles



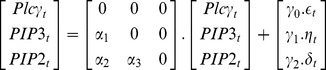
(22)


In a recent study [7], signalling mechanisms between 11 molecules were inferred from single-cell data using flow-cytometry in conjunction with Bayesian network structure learning algorithms. The resulting network was shown to validate existing associations as well as discovering novel undocumented associations. Of interest, was the sub-network consisting of three molecules 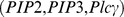 weakly connected to the rest of the molecules in the network (see Fig. 3 in [7]). The network structure inferred from the molecular expression data between these three molecules 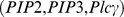 consisted of the following directed edges 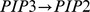, 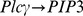 and 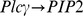. A quick inspection would reveal the resemblance of the relationships between these three molecules (22) to that of coherent Type-I FFL motif (Fig. 1c, Case 3) discussed earlier. The expected and the inferred relationships along with the influence paths for these three molecules can be found in (Table 3, Sachs et al., 2005). While the authors acknowledged that the directionality between (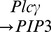, *recruitment leading to phosphorylation*) inferred from the data was opposite to that established in the literature [43] (see Supplementary Material, Table I, Sachs et al., 2005), they successfully validated (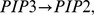 *precursor-product*) and (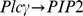, *direct hydrolysis to IP3*) [44], [45] (see Supplementary Material, Table I, Sachs et al., 2005). While several data sets were investigated in [7], we restrict the present study to the unperturbed data set comprising the expression of 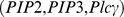 across 853 single cells. Prior to investigating the impact of noise on the network inference between the three molecules, we found the distribution of the expression levels across the single-cells to be positively skewed, indicating large variations in the expression estimates across the cells. Interestingly, we also found the variance in the expression levels proportional to their average value across the molecules 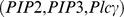. Box-Cox [46] transforms are widely used in literature to minimize the skew in the distribution and suppress non-constant variance as a function of magnitude. In the present study, we used the log-transform which is the limiting case of the classical Box-Cox transform to minimize the skew in the distribution of the expression across these three molecules. Therefore, the results across the raw as well as the log-transformed data are presented.

Three different networks 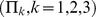 were investigated. 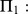 Network inferred from the given data; 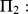 Network inferred from data generated from the linear model (22) fit to the given data without any constraints on the model parameters; 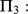 network inferred from data generated by the linear model fit (22) to the given data with constraint on the noise variance to be equal 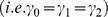. The above exercise was repeated for the raw as well as the log-transformed protein expression data and the corresponding edge confidences were estimated. The approach is outlined below.

- **Step 1**: Given the expression 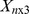 of the three molecules across 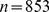 cells.
- **Step 2**: Generate independent realizations 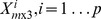 by resampling 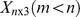 with replacement. In the present study, we set 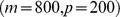. Set each column in 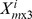 to zero-mean.
- **Step 3**: Set 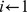.
- **Step 4**: Infer the network structure from 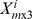 using the GS algorithm. Let the resulting network be 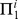.
- **Step 5**: Estimate the parameters (i.e. regression coefficients and noise variances (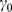, 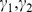) by fitting the linear model (22) to 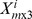. Generate 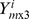, using the estimated model parameters and zero-mean i.i.d. noise terms 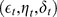 sampled from a log-normally distributed noise to accommodate for the positive-skew in the distribution. Infer the network structure from 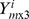 using the GS algorithm. Let the resulting network be 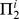.
- **Step 6**: Generate data 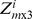, using the linear model in Step 5 with the additional constraint on equal noise variance 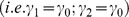 in (22). Infer the network structure from 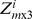 using the GS algorithm. Let the resulting network be 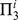.
- **Step 7**: Set 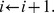

- **Step 8**: Repeat Steps 4–7 till 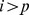.
- **Step 9**: Estimate the confidences of the edges for each of the networks 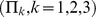.
- **Step 10**: Repeat Steps 1–9 for the log-transformed data with normally distributed noise as opposed to log-normally distributed noise in Steps 5 and 6.

#### Raw Data

**Figure 4:**
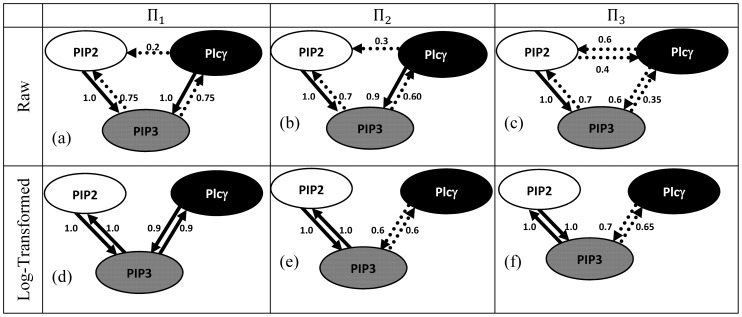
Bayesian networks inferred using Grow-Shrink algorithm from the molecular expression data (PIP2, PIP3, Plcγ) with sample-size 800 and Pearson correlation (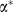 = 0.01) are shown in (a–f). Confidences estimated from 200 independent bootstrap realizations are shown along the edges. Edges with 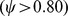 are shown by solid arrows whereas others 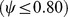 are shown by dotted arrows. Edges with confidence 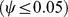 are deemed noisy and excluded for clarity. The edge confidences of the networks 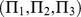 inferred from the raw data are shown in (a), (b) and (c) respectively. Those inferred on the log-transformed data are shown in (d), (e) and (f) respectively.

The networks 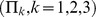 inferred using the raw data for the molecules 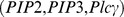 are shown in Figs. 4a–4c respectively. Network structures inferred from the raw data (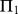, Step 4) and those of the linear model fit (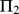, Step 5) exhibited considerable similarity as reflected by their edge confidences, Figs. 4a and 4b. The confidence was high along 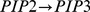 and 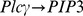, and markedly low along 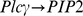, Figs. 4a, 4b. Noise variance estimated from the linear model fit (Step 5) of the raw data revealed around a two-fold difference 
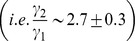
. Constraining the noise variance to be equal 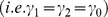 had a marked effect on the resulting network (Step 6) 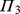, Fig. 4c. The edge confidences were considerably high along 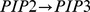 as seen earlier (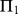 and 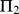), Figs. 4a, 4b. However, relatively smaller edge confidence along between (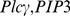)along either directions, Fig. 4c, in contrast to Figs. 4a or 4b was also observed. More importantly, constraining the noise variance also increased the edge confidences between (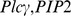) along either directions in contrast to those shown in Figs. 4a and 4b (i.e. 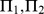). Thus forcing the noise variance to be equal had a pronounced effect on the inferred network.

#### Log-transformed Data

In order to minimize the impact of skewness on the conclusions, the entire exercise was repeated on the log-transformed data. The resulting networks along with confidence of the edges are shown in Figs. 4d–4f. The networks 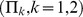 inferred from the log-transformed data (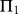, Step 4) and those from data generated on the linear model fit (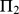, Step 5) along with the edge confidences are shown in Figs. 4d–4e respectively. The noise variance estimates from the linear model fit to the log-transformed data revealed no marked difference 
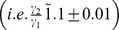
 in contrast to what was observed in the raw data. Since there were no marked discrepancies in noise variance, forcing the noise variance to be equal 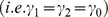 had no profound effect on the resulting network (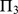, Step 6) Fig. 4f as expected. This was revealed by the similar edge confidences across 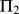 and 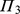. Furthermore, it is important to note that the networks 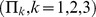 inferred from the log-transformed data unlike those from raw data, failed to capture any relationship 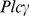 and 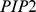.

## Discussion

Real-world entities work in concert as a system and not in isolation. Associations between such entities are usually unknown. Inferring associations and network structure from data obtained across the entities is of great interest across a number of disciplines. The recent surge of high-throughput molecular assays in conjunction with a battery of algorithms has facilitated validating established associations while discovering new ones with the potential to assist in novel hypothesis generation. These associations and networks have been shown to capture possible causal relationships under certain implicit assumptions and proven to be useful abstractions of the underlying signaling mechanism. Such an understanding can provide system level insights and often precedes developing meaningful interventions. Several network inference algorithms have been proposed in literature including those that depend on pairwise and conditional dependencies. However, little attention has been given to the impact of possible discrepancies in noise variance across the data obtained across the molecular entities. In molecular settings, such discrepancies can be attributed to several factors including inherent stochastic mechanisms, heterogeneity in cell populations, variations in abundance of the molecules, variation in binding affinities, sensitivity of the measurement device and other experimental artifacts. Understanding the discrepancies in noise variance is critical in order to avoid spurious conclusions and an important step prior to identifying the source of the noise.

The present study clearly elucidated the non-trivial impact of discrepancies in noise variance on associations and network inference algorithms across synthetic as well as experimental data. The impact of large discrepancies in noise variance on associations and network structure inferred from data generated using linear models of popular network motifs and fundamental connections as well as those from experimental protein expression profiles were investigated. Analytical expressions and simulations were presented elucidating the non-trivial impact of noise on three popular molecular network motifs and fundamental connections (common effect, three-chain and coherent Type-I feed-forward loop). It was shown that discrepancies in noise variance can significantly alter the results of pairwise dependencies, conditional dependencies as well as constraint-based Bayesian network structure learning techniques that implicitly rely on tests for conditional independence. As expected, the discrepancies in noise variances was found to result in markedly different topologies from those of their noise free counterpart challenging reliable inference of the underlying network topology. Such discrepancies were also shown to result in spurious conclusion of similar structures across markedly distinct network topologies. The impact of discrepancies in noise variance were also investigated on publicly available single-cell molecular expression profiles of a sub-network comprising of three molecules (PIP2, PIP3, Plcγ) involved in human T-cell signaling. The sub-network shared considerable resemblance to the coherent Type-I feed-forward loop. The distribution of the raw expression estimates across these three molecules was positively skewed indicating large variations in the expression estimates across the single-cells. Variance about the average expression across the three molecules was found to be markedly different and proportional to their average values. Several factors can contribute to such discrepancies including: abundance of these molecules, antibody binding characteristics, uncertainty due to possible overlap in the wavelengths corresponding to the colors tagged to the molecules. In the present study, a linear model was fit to the molecular expression data. Parameter estimates from the linear model indicated significant discrepancies in the noise variances across the molecules. Adjusting for these discrepancies in the model was shown to significantly affect the edge confidences of the resulting networks, hence the topology. The results were presented on the raw molecular expression data as well as its log-transformed counterpart. As expected, log-transforming the data not only reduced the positive skew of the expression profile but also rendered the noise variance estimates comparable across the molecules. However, the networks inferred using the log-transformed data were considerably different from those inferred on the raw data. While identifying the source of the variation and controlling for the same prior to the network inference may be the long-term goal and a research problem in its own merit, understanding the impact of discrepancies in noise variance is a critical step in this direction. While the present study focused on simple network motifs comprising of three molecules, the concerns are likely to be aggravated across more complex network topologies. The analytical treatment provided in the present study has the potential to be translated across other setting such as genome-wide association studies (GWAS) [47]. Unlike the molecular network motifs investigated in this study, GWAS investigate the impact of causal genes and variants on a given trait or set of traits. Similar to the concerns presented in the present study, discrepancies in biological variances across the traits is not uncommon and can have a pronounced effect in discerning the relationship between the causal and the traits. However, given the intricacies accompanying GWAS studies a more detailed investigation is required.
